# Targeting intercellular adhesion molecule-1 prolongs survival in mice bearing bevacizumab-resistant glioblastoma

**DOI:** 10.18632/oncotarget.18859

**Published:** 2017-06-29

**Authors:** Yuji Piao, Verlene Henry, Ningyi Tiao, Soon Young Park, Juan Martinez-Ledesma, Jian Wen Dong, Veerakumar Balasubramaniyan, John F. de Groot

**Affiliations:** ^1^ Department of Neuro-Oncology, The University of Texas MD Anderson Cancer Center, Houston, Texas, USA; ^2^ Department of Neuro-Surgery, The University of Texas MD Anderson Cancer Center, Houston, Texas, USA

**Keywords:** intercellular cell adhesion molecule-1, bevacizumab, signal transducer and activator of transcription 3, macrophage

## Abstract

Intercellular cell adhesion molecule 1 (ICAM-1; also known as CD54) is overexpressed in bevacizumab-resistant glioblastoma. In the present study, we tested our hypothesis that highly expressed ICAM-1 mediates glioblastoma’s resistance to antiangiogenic therapy. We validated ICAM-1 overexpression in tumors resistant to antiangiogenic therapy using real-time polymerase chain reaction, immunohistochemistry, and Western blotting. We also detected ICAM1 expression in most glioma stem cells (GSCs). We investigated the mechanism of ICAM-1 overexpression after bevacizumab treatment and found that ICAM-1 protein expression was markedly increased in a time-dependent manner in GSC11 and GSC17 cells under hypoxic conditions *in vitro*. We also found that hypoxia induced ICAM-1 overexpression through the up-regulation of phosphorylated signal transducer and activator of transcription (p-STAT3). Hypoxia-induced p-STAT3 increased the mRNA transcription of ICAM-1, which we could inhibit with the STAT3 inhibitor AZD1480. Next, we used GFP-tagged ICAM-1 shRNA lentivirus to knock down ICAM-1 in GSC11 and GSC17 glioma cell lines. Then, we injected shICAM-1 GSC11 and scramble glioma stem cells into the brains of nude mice. Mice bearing tumors from shICAM-1 GSC11 cells survived significantly longer than mice injected with control cells did. The tumor sizes was significantly decreased in mice bearing tumors from shICAM-1 cells than that in mice bearing tumors from GFP-tagged GSC11 control cells. Knocking down ICAM-1 suppressed tumor invasion *in vitro* and *in vivo* and inhibited macrophage infiltration to the tumor site in bevacizumab-treated mice. Our findings suggest that ICAM-1 is a potentially important mediator of tumor migration and invasion in bevacizumab-resistant glioblastoma. Targeting ICAM-1 may provide a new strategy for enhancing the efficacy of antiangiogenic therapy against glioblastoma and preventing the invasive phenotype of the disease.

## INTRODUCTION

Glioblastoma is the most common and aggressive human primary brain tumor. Patients with the disease, even those who have received multiple standard treatments including ionizing radiation and chemotherapy, have a median survival time of less than 15 months [1]. Effective approaches that increase the survival rate and life quality of glioblastoma patients are urgently needed.

The antiangiogenic agent bevacizumab (Avastin; Roche/Genentech, South San Francisco, CA), a recombinant humanized monoclonal antibody directed against vascular endothelial growth factor (VEGF), has been investigated in the treatment of glioblastoma [2]. Bevacizumab, either alone or with chemotherapy, has been shown to suppress glioblastoma progression by inhibiting tumor angiogenesis and/or tumor growth. However, after initially responding to bevacizumab treatment, glioblastoma develops resistance to the drug, becoming increasingly invasive and growing faster [3]. Moreover, several studies have shown that glioblastoma develops resistance through multiple mechanisms, such as the enhancement of alternative pro-angiogenic signaling pathways, recruitment of bone marrow-derived pro-angiogenic cells to the tumor, increasing of pericyte coverage of the tumor vasculature, and promotion of tumor invasion [4].

To elucidate the mechanism of glioblastoma’s resistance to antiangiogenic therapy, we performed a gene expression profiling analysis of GSC11 glioblastoma xenografts from mice treated with or without bevacizumab. We found that the expression of intercellular adhesion molecule-1 (ICAM-1, also known as CD54) in glioblastoma tumors from bevacizumab-treated mice was higher than that in the glioblastoma tumors from vehicle-treated control mice. ICAM-1, a member of the immunoglobulin supergene family, is constitutively localized on the cell surface. ICAM-1 contains five extracellular immunoglobulin-like domains that function in adhesive cell-cell or cell-matrix interactions by binding to two integrins belonging to the β_2_ subfamily: lymphocyte function-associated antigen-1 (LFA-1; also known as CD11α/CD18) and macrophage antigen-1 (Mac-1; also known as CD11b/CD18) [5]. ICAM-1 is abundantly expressed in a variety of cell types, including fibroblasts, leukocytes, keratinocytes, and endothelial cells [6]. Importantly, ICAM-1 is markedly expressed in many different types of human cancer cells, including lung, pancreatic, breast, and prostate cancer cells, as well as in glioma [7].

We hypothesized that highly expressed ICAM-1 partly mediates glioblastoma’s resistance to antiangiogenic therapy. To test this hypothesis, we investigated the regulation of ICAM-1 expression in bevacizumab-treated glioma stem cells (GSCs) *in vitro* and in *in vivo* murine glioma models. Our findings provide new insight into the resistance of antiangiogenic therapy and a new strategy for improving the efficacy of antiangiogenic therapy in glioblastoma patients.

## RESULTS

### ICAM-1 is overexpressed in bevacizumab-resistant GSC11 xenografts

**Figure 1:**
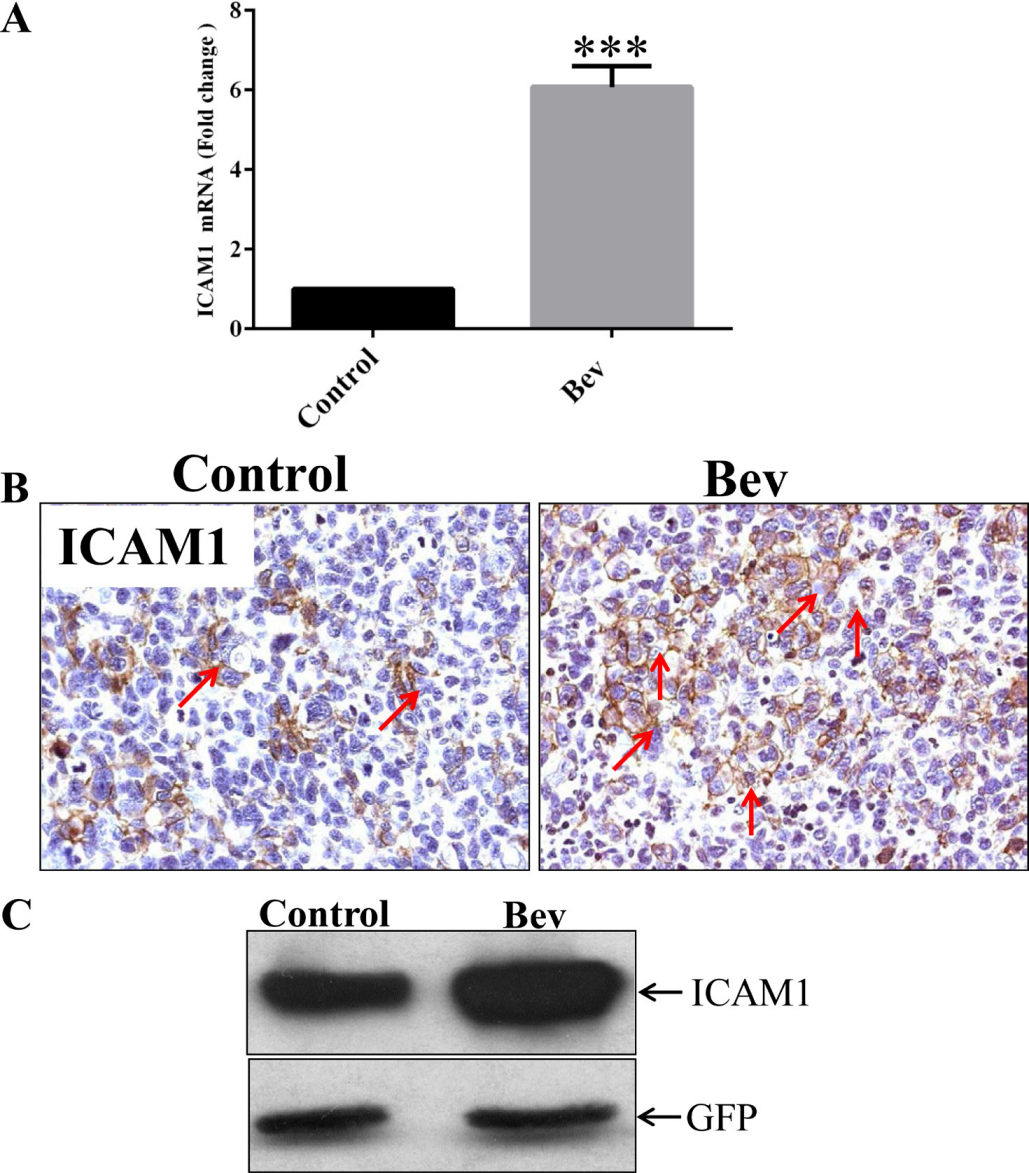
ICAM-1 is overexpressed in bevacizumab-resistant GSC11 xenografts (**A**) Quantitative real-time PCR, in which GAPDH mRNA expression was used as internal control, revealed that the fold-change in the RNA expression level of ICAM-1 in xenografts from bevacizumab-treated mice was significantly higher than that in vehicle-treated control mice. (**B**) Immunohistochemical analysis revealed that GSC11 xenografts from mice treated with vehicle only (Control) had lower ICAM-1 expression than those from mice treated with bevacizumab (Bev) did. Representative images are shown; cell membranes expressing ICAM-1 are brown (indicated by red arrows; magnification × 400). (**C**) Western blotting revealed that ICAM-1 was expressed in the GSC11 xenografts regardless of whether mice were treated with vehicle (Control) or bevacizumab (Bev).

In a previous study, we found that bevacizumab prolonged the survival of GSC11 xenograft–bearing mice; however, after 5 weeks of bevacizumab treatment, the mice developed resistance to the antiangiogenic therapy and died [8]. To identify the molecular mechanism underlying gliobastoma resistance to anti-VEGF therapy, we treated GSC11 glioma–bearing mice with or without bevacizumab until the mice became moribund (2 mice per group). We extracted RNA from the xenograft tumors and subjected them to gene expression microarrays. Our analysis of Affymetrix gene expression profiling data revealed that ICAM-1 expression in the bevacizumab-treated tumors was significantly higher than that in the untreated control tumors. We validated this result with real-time polymerase chain reaction (PCR), which revealed that ICAM-1 expression in the bevacizumab-treated xenograft tumors was 7-fold higher than that in the vehicle-treated control tumors (*p* < 0.001; Figure 1A). Immunohistochemistry and Western blotting further validated these findings, showing that ICAM-1 protein expression was significantly enhanced in the bevacizumab-treated tumors compared with that in the control tumors (Figure 1B and Figure 1C).

### Hypoxia induces ICAM-1 overexpression through phosphorylated signal transducer and activator of transcription factor 3 (STAT3)

**Figure 2:**
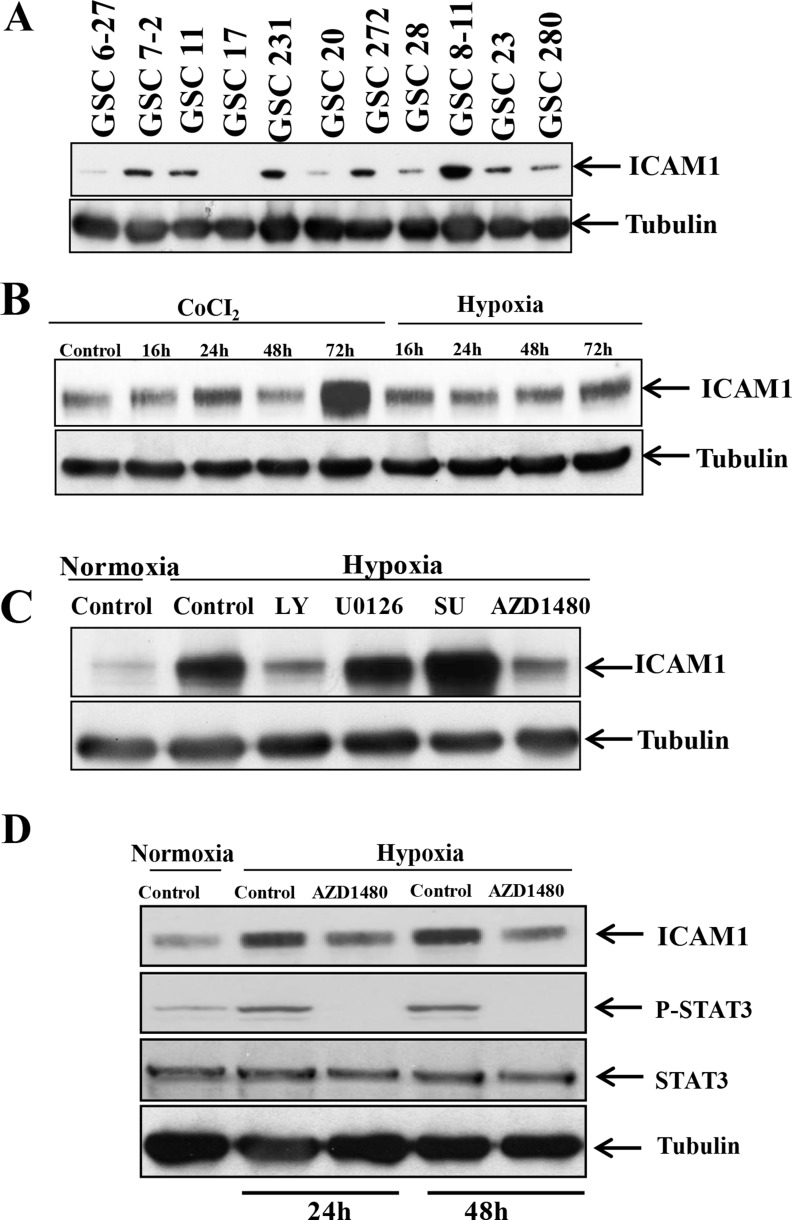
p-STAT3 upregulates ICAM-1 expression in GSC11 cells under hypoxic conditions (**A**) Western blotting revealed that ICAM-1 is expressed in multiple human glioma stem cell lines. (**B**) Western blotting revealed that the ICAM-1 expression level in GSC11 cells increased in a time-dependent manner under hypoxic conditions. (**C**) Western blotting revealed that ICAM-1 was expressed in GSC11 cells treated with or without LY, U0126, SU, or AZD1480 under hypoxic conditions. (**D**) Western blotting revealed the p-STAT3 expression level in the nuclear fraction of GSC11 cells treated with or without AZD1480 under hypoxic conditions.

We performed Western blotting to determine whether ICAM-1 is expressed in a panel of glioma stem cells. We found that ICAM-1 was significantly overexpressed in GSC6-27, GSC7-2, GSC11, GSC231, GSC20, GSC272, GSC28, GSC8-11, GSC23, and GSC280 cells and detectable in GSC17 cells (Figure 2A). Next, we sought to identify the factors that results in the induction of ICAM1 expression in glioma stem cells. In a previous study, we found that antiangiogenic therapy can reduce the blood supply within glioblastoma, leading to a more hypoxic tumor microenvironment [9]. Therefore, we hypothesized that hypoxia inducible factor-1α (HIF-1α) up-regulates ICAM-1 expression in glioma cells. To test this hypothesis, we collected GSC11 glioma xenograft tumors from mice treated with or without bevacizumab. Glioma tumors were embedded in paraffin block, and subjected to co-immunofluorescence staining for HIF-1α and ICAM-1. ICAM-1 was highly co-expressed in HIF-1α positive areas on the tumor cell surface. We also found that the protein expression levels of HIF-1α and ICAM-1 in bevacizumab-treated tumors were higher than those of vehicle-treated control tumors (Supplementary Figure 1).

To ascertain whether ICAM-1 expression is higher under hypoxic conditions compared to normoxia, we incubated GSC11 cells under hypoxic conditions or treated the cells with CoCl_2_ and then performed Western blotting for ICAM-1. We found that the ICAM-1 expression level increased in a time-dependent manner under hypoxic conditions and after CoCl_2_ treatment (Figure 2B). We confirmed this result using GSC17 glioma stem cells (as GSC17 cells have a lower baseline ICAM1 expression level) and found that the ICAM-1 expression level was also increased under hypoxic conditions or after CoCl_2_ treatment in these cells (Supplementary Figure 2). To identify the molecular pathway underlying the increase in ICAM-1 expression under hypoxic conditions, we treated GSC11 cells with inhibitors of phosphoinositide-3 kinase (LY294002; 30 nM), MEK (U01260; 10 nM), Src (SU6656; 4 µM) and signal transducer and activator of transcription factor 3 (STAT3) (AZD1480; 5 µM) under hypoxic conditions. We found that treatment with the phosphoinositide-3 kinase inhibitor or the STAT3 inhibitor blocked ICAM-1 expression (Figure 2C).

Because STAT-3 is known to activate and stabilize HIF-1α, we also sought to determine whether the phosphorylated STAT3 (p-STAT3) expression level is also increased under hypoxic conditions. We obtained nuclear protein extracts from GSC11 cells under hypoxic conditions for 2 days and then assessed their p-STAT3 expression levels. We found that the p-STAT3 expression level is increased under hypoxic conditions. The ICAM-1 expression level in cells treated with the STAT3 inhibitor AZD1480 under hypoxic conditions was lower than that in control cells (Figure 2D).

### Knockdown of ICAM-1 prolonged survival and reduced glioma tumor volume in mice treated with bevacizumab

**Figure 3:**
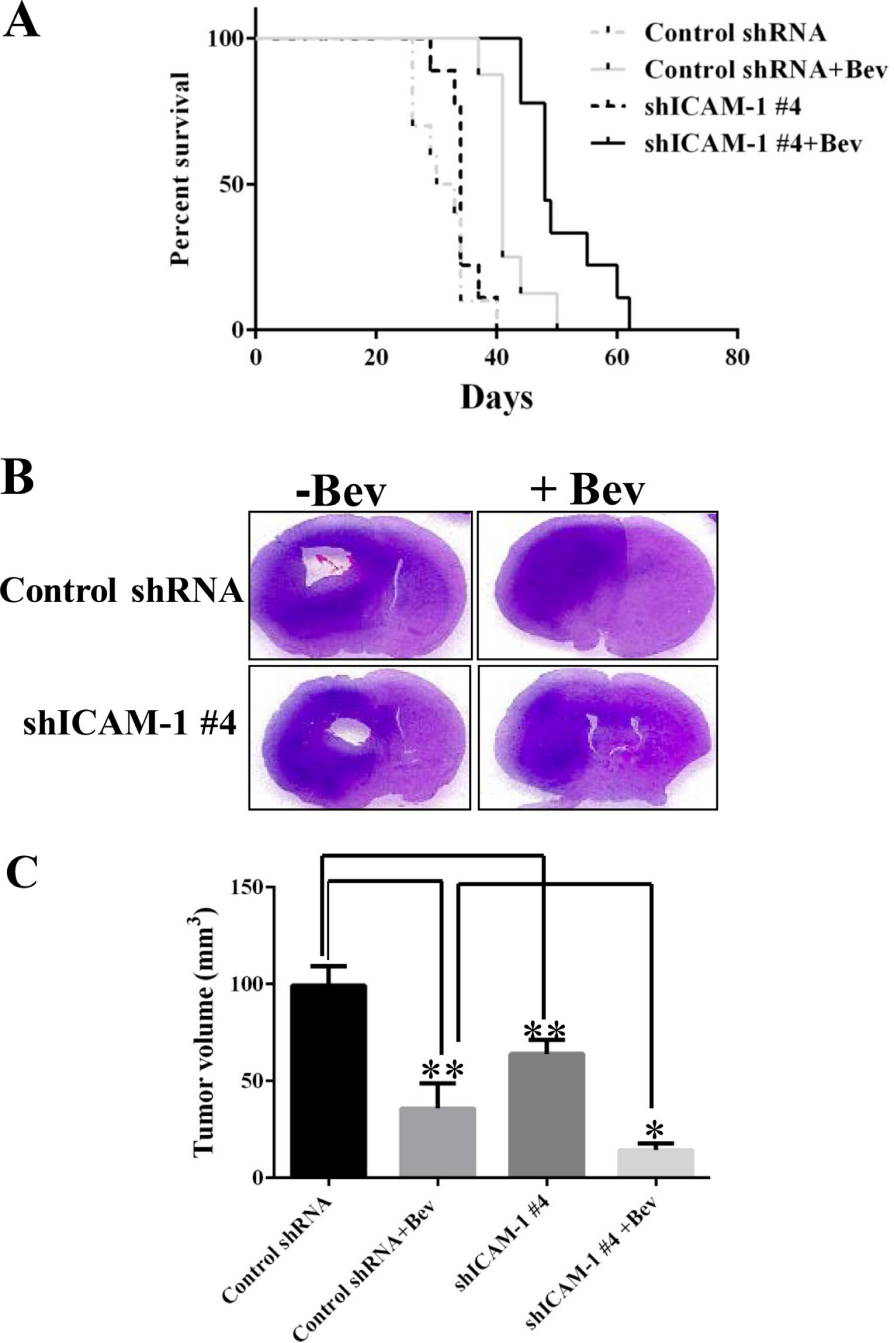

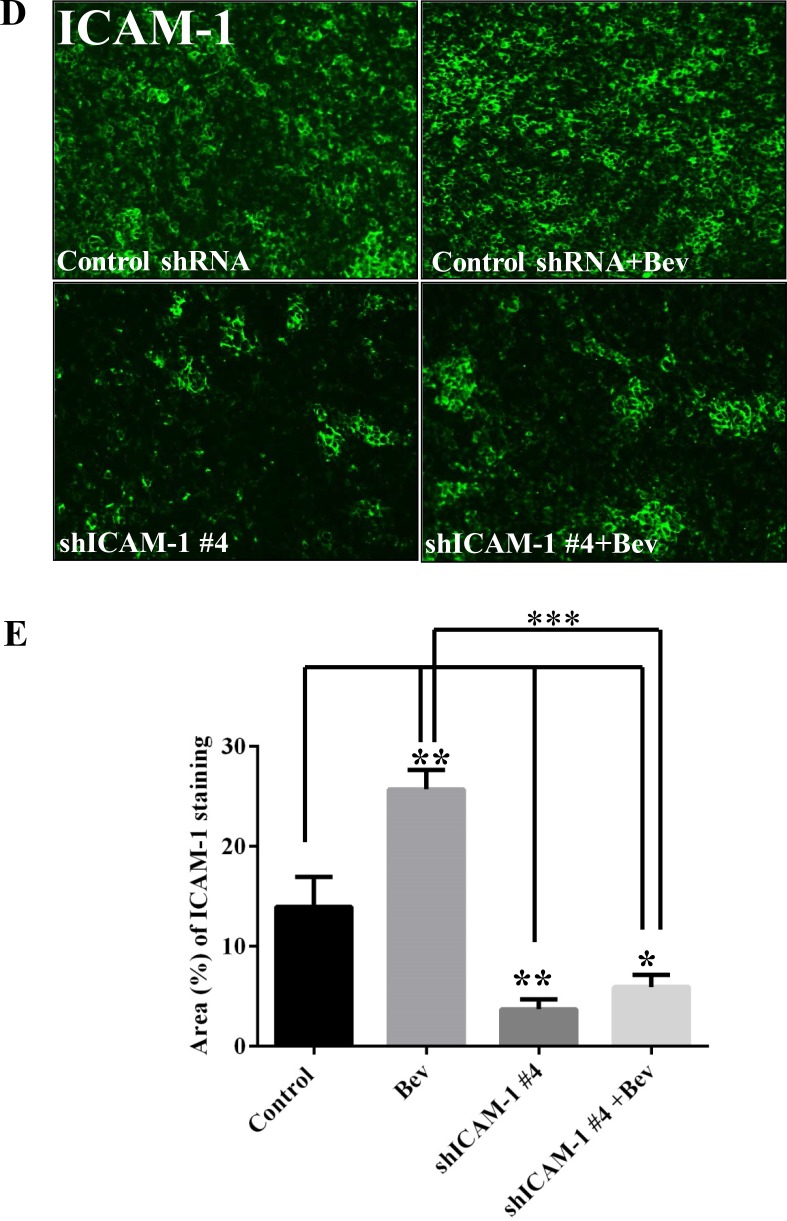
ICAM-1 knockdown prolongs survival in mice with bevacizumab-resistant glioblastoma (**A**) Kaplan-Meier survival analysis revealed that when mice bearing shRNA ICAM-1 #4 xenografts prolonger survival compared with the mice bearing GSC11 GFP after treatment with bevacizumab. H&E staining analysis (**B**) and the bar graph of tumor volume (**C**) revealed that the size of the tumor which from mice bearing shRNA ICAM-1 #4 xenografts significantly decreased compared with the tumor from the mice bearing GSC11 GFP after with or without treatment of bevacizumab for 5 weeks. Representative images are shown (**D**) for the immunofluorescence staining of ICAM-1 (green) revealing that ICAM-1 expression was significantly lower in tumor from mice bearing shRNA ICAM-1 xenografts compared with tumor from mice bearing GSC11GFP after treatment with or without bevacizumab. Representative photomicrograph images are shown (magnification × 200). (**E**) The bar graph from the percentage of ICAM-1 staining in tumor area (D) revealed that ICAM-1 expression was blocked in tumor from mice bearing shRNA ICAM-1#4 compared with tumor from the mice bearing GSC11 GFP (^*^P < 0.05; ^**^P < 0.01). Representative photomicrograph images are shown (magnification × 200).

To determine whether ICAM-1 could serve as a therapeutic target in glioblastoma, we used five independent shRNAs to stably knock down ICAM-1 expression in GSC11 glioma stem cells. We found that the shICAM-1 #4 clone expressed the lowest level of ICAM-1 (Supplementary Figure 3) which was then used for additional *in vivo* studies. We next determined whether knocking down ICAM-1 prolongs the survival of mice that receive anti-VEGF therapy. Glioma cells from the shICAM-1 #4 clone were expanded and then injected orthotopically into nude mice that did or did not receive bevacizumab treatment. We separated 10 mice per group, total four groups. The median survival times of the untreated shRNA control mice and the bevacizumab-treated shRNA control mice were 30 days and 41 days. However, the median survival time of the bevacizumab-treated, shICAM-1 #4 tumor–bearing mice (48 days) was significantly longer than that of the vehicle-treated shRNA control tumor–bearing mice (*p* < 0.0001) and that of the bevacizumab-treated shRNA control tumor–bearing mice (*p* < 0.01) (Figure 3A).

Mouse brain sections were stained with hematoxylin and eosin (H&E). Tumors with lower ICAM-1 expression had a significantly reduction in tumor size (Figure 3B). At 5 weeks, the mean tumor volume of the shRNA control mice (99.27 ± 10.04 mm^3^) was significantly larger than those of the bevacizumab-treated shRNA control mice (12.88 ± 10.46 mm^3^; *p* < 0.01) and shICAM-1 #4 mice (*p* < 0.01). More importantly, the mean tumor volume of the bevacizumab-treated shICAM-1 #4 mice (14.35 ± 3.50 mm^3^) was significantly smaller than that of the bevacizumab-treated shRNA control mice (*p* < 0.05) and vehicle-treated control shRNA mice (*p* < 0.001) (Figure 3C). Small tumor volume correlated with low ICAM-1 expression level (as assessed by immunofluorescence) in the shICAM-1 #4 group compared with the parental GSC11 group (Figure 3D, 3E).

### ICAM-1 knockdown suppresses glioma cell invasion

**Figure 4:**
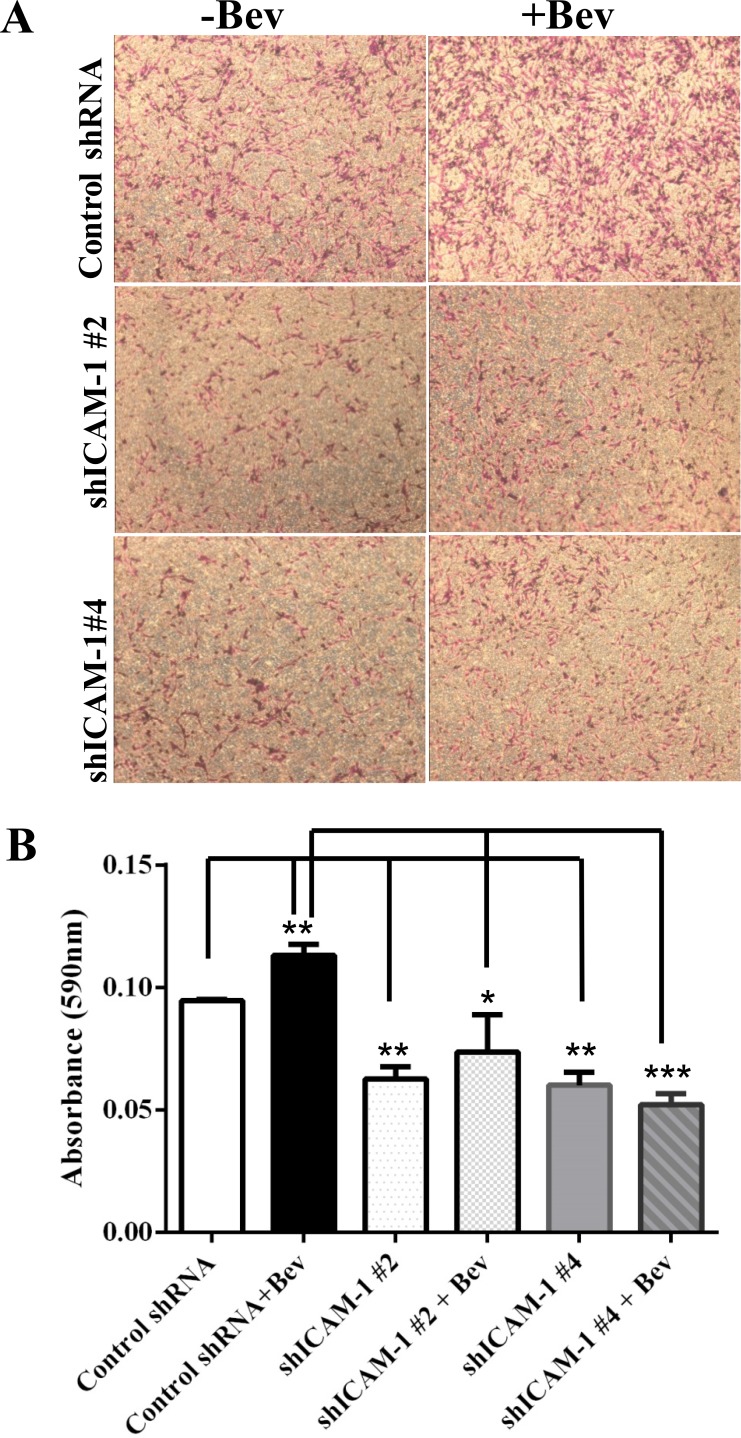

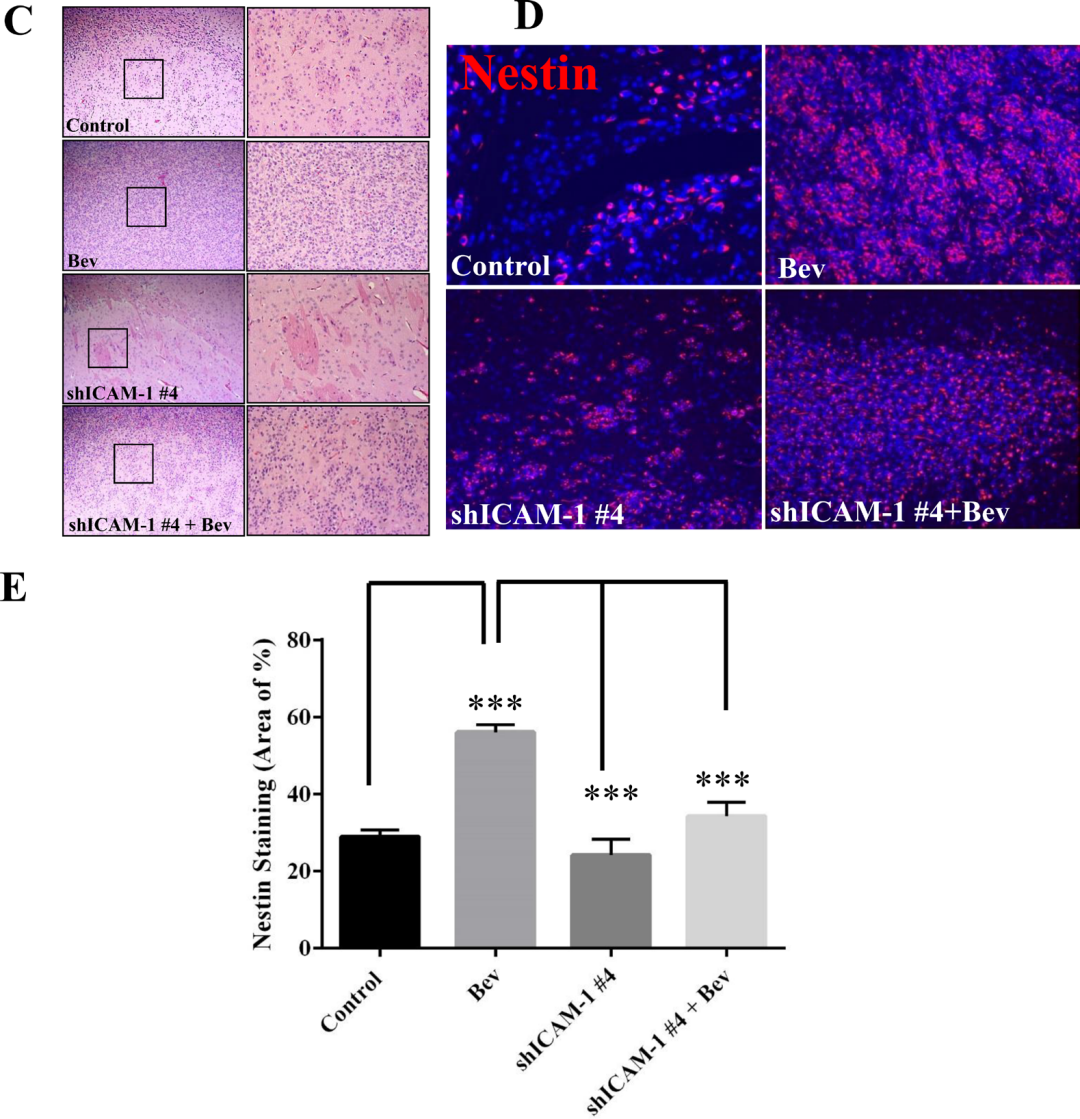
ICAM-1 knockdown suppresses cell invasion *in vitro* and *in vivo* (**A**) and (**B**) GSC11GFP control and shRNA ICAM-1#4 cells treated with or without bevacizumab were subjected to a transwell migration assay. Photomicrographs of representative samples from the assay (A) and the bar graph of quantitative absorbance (590 nm) (B) revealed that shRNA ICAM-1#2 and shRNA ICAM-1 #4 cells significantly were less invaded compared with GSC GFP after cells treated with or without bevacizumab. Representative photomicrographs from three independent experiments are shown (magnification ×200) (^*^P < 0.05; ^**^P < 0.01). (**C**) H&E staining analysis revealed that the shRNA ICAM-1#4 derived tumors had a lower invasive capacity compared with the GSC11GFP derived tumors after with or without bevacizumab treatment. The square area indicates the areas of tumor invasion, which are shown at right (magnification ×200). (**D**) and (**E**) Immunofluorescence staining for Nestin (red) (D) and the bar graph showing the percentage of Nestin-positive staining in the areas of tumor invasion (E) revealed that shRNA ICAM-1#4 derived tumor had lower Nestin expression in areas of tumor invasion compared with GSC11 GFP derived after mice were treated with or without bevacizumab.

Anti-VEGF therapy has been shown to increase the invasiveness of glioma xenografts in mice [9]. To determine whether ICAM-1 is involved in this tumor invasion phenotype, we subjected shICAM-1 #2, and shICAM-1 #4 cells to a transwell invasion assay *in vitro*. We found that, compared with their parental control GSC11 cells, the shICAM-1 #2 and shICAM-1 #4 cells had a significantly lower invasive capacity without bevacizumab treatment (Figure 4A). Quantification analysis of the absorbance at 595 nm revealed that the cell invasion of the bevacizumab-treated parental GSC11 cells was significantly higher than that of the vehicle-treated parental GSC11 cells (19% ± 6%; *p* < 0.01). However, the cell invasion of the bevacizumab-treated shICAM-1 #2 cells (34.40% ± 16.00%; *p* < 0.05) and bevacizumab-treated shICAM-1 #4 cells (53.95% ± 4.21%; *p* < 0.001) were both significantly less than that of the bevacizumab -treated parental GSC11 cells (Figure 4B).

We previously found that anti-VEGF therapy increases the invasive characteristics of GSC11 cells *in vivo* [9]; that is, tumor cells that develop bevacizumab resistance become more aggressive and more invasive in the setting of continuous bevacizumab treatment. However, as described above, we also found that after treated with bevacizumab, glioma cells with ICAM-1 knockdown were less invasive than bevacizumab-treated parental glioma cells (Figure 4C, 4D). Glioma xenograft tumors were stained for the glioma stem cell marker Nestin. The data showed that the percentage of Nestin-positive tumor in the vehicle-treated control mice was 28.90 ± 1.76%, compared to 56.07 ± 1.87% of cell in tumors exposed to bevacizumab treatment (vs Control: *p* < 0.001). However, the percentage of Nestin-positive tumor area markedly decreased in shICAM-1 tumor–bearing mice treated with bevacizumab (34.20 ± 3.66%, *p* < 0.001) or vehicle (24.10 ± 4.16 %, *p* < 0.001), compared to GSC11GFP generated tumor treated with bevacizumab respectively (Figure 4E). These finding suggest that knockdown ICAM-1 expression in tumor cells reduced anti-VEGF therapy associated glioma cell invasion *in vitro* and *in vivo*.

### ICAM-1 knockdown suppresses macrophage infiltration of the tumor site

**Figure 5:**
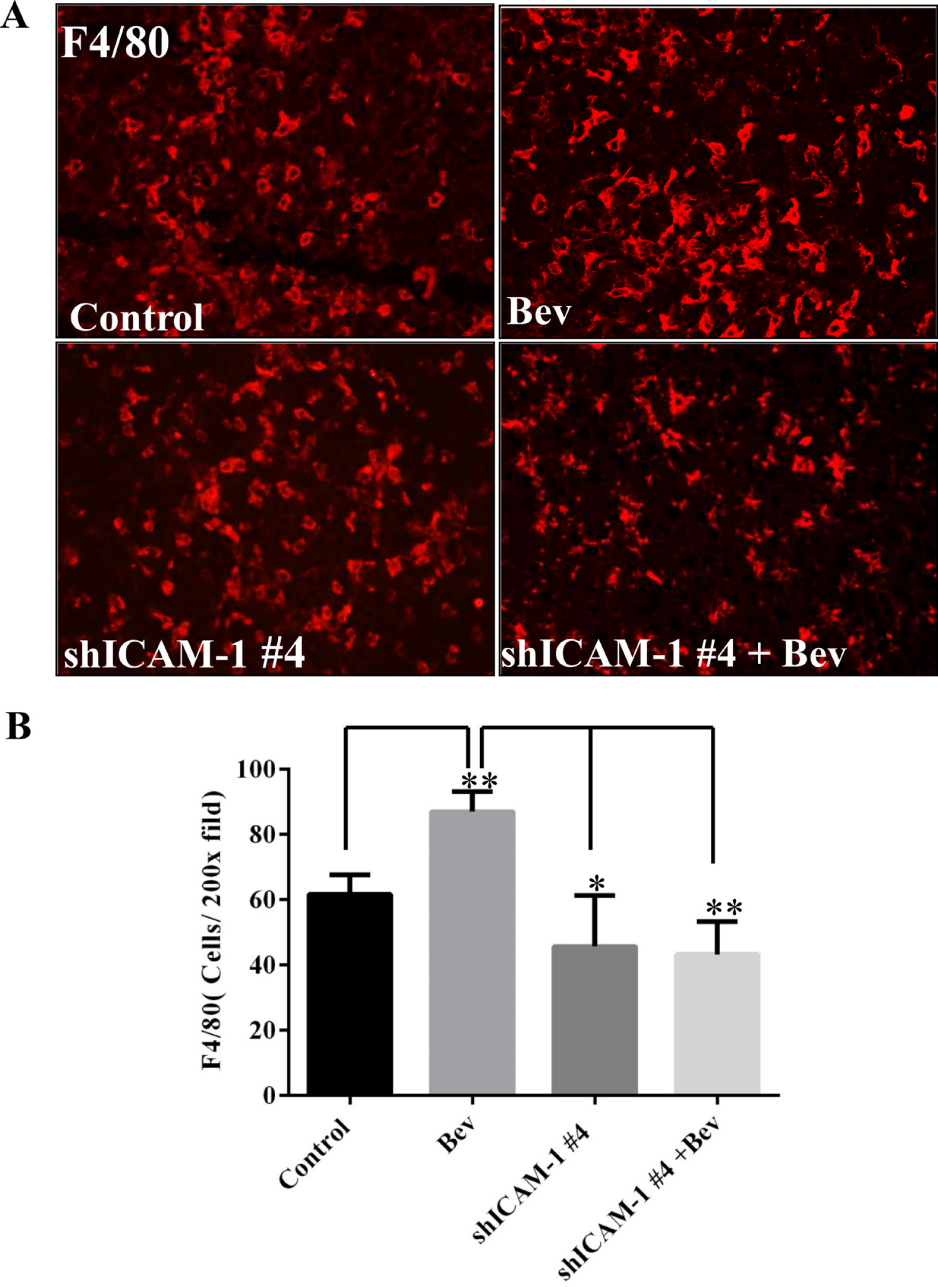
ICAM-1 knockdown inhibits macrophage infiltration into tumor in bevacizumab-treated mice (**A**) and (**B**) Immunofluorescence staining with F4/80 (red) (A) and the bar graph of F4/80 positive cells in each high powered microscopic field (B) revealed that shRNA ICAM-1generated tumor had less F4/80 positive macrophage infiltration compared with GSC11GFP generated tumor. Representative photomicrographic images are shown (magnification ×200). (**C**) Immunofluorescence staining for Nestin (green) and F4/80 revealed that blocking ICAM-1 by treatment with ICAM-1 antibody 10 µg/ml or 20 µg/ml in GSC11 cells reduced GSC11 binding to macrophages. (**D**) The bar graph represents the ratio of Nestin positive to F4/80 positive. Data showed that blocking ICAM-1 decreased GSC capacity of binding to macrophages.

ICAM-1 plays a key role in regulating myeloid cell migration by binding with LFA-1 and Mac-1, both of which are expressed on leukocytes and promote these cells’ adhesion and transendothelial migration. In a previous study, we found that macrophages infiltrated the tumor site in the setting of bevacizumab resistance [9]. We hypothesized that blocking ICAM-1 interferes with macrophage migration to the tumor, thereby prolonging mouse survival. To confirm this hypothesis, we performed an immunofluorescence experiment on tumors treated *in vivo* as previously described above. We found that the percentage of F4/80-positive tumor in the bevacizumab treatment was 87.00 ± 6.24%, compared to 61.67 ± 6.02% vehicle-treated control mice (vs Control: *p* < 0.01). However, the percentage of F4/80-positive tumor area significantly decreased in shICAM-1 tumor–bearing mice treated with bevacizumab (43.33 ± 10.07%, *p* < 0.01) or vehicle (45.67 ± 15.63%, *p* < 0.05), compared to GSC11GFP generated tumor treated with bevacizumab, respectively (Figure 5A).

To further elucidate the relationship between ICAM-1 expression in tumor cells and macrophage infiltration, we performed an *in vitro* cell binding assay. We treated GSC11 cells with or without an anti–ICAM-1 antibody (10 µg/ml or 20 µg/ml) co-cultured with macrophages. We stained GSC11 cells with Nestin (green) and macrophages with F4/80 (red). We found that untreated GSC11 cells were adherent to macrophages; the ratio of Nestin positive cells to F4/80 positive cells was 0.82 ± 0.03. However, GSC11 treated with the anti–ICAM-1 antibody (10 ng/ml or 20 ng/ml) reduced this ratio to 0.57 ± 0.08 (*p* < 0.01) and 0.52 ± 0.06 (*p* < 0.01), respectively, a significant inhibition of GSC11 cells adherent to macrophage (Figure 5B).

## DISCUSSION

In the present study, we found that ICAM-1 is overexpressed in glioma that has developed resistance to antiangiogenic therapy. To our best knowledge, our study is the first to demonstrate that ICAM-1 overexpression is correlated with resistance to antiangiogenic therapy in glioma.

We found that ICAM-1 is expressed in several glioma stem cell lines and that ICAM-1 knockdown increases glioma cells’ sensitivity to antiangiogenic therapy. More importantly, we found that mice bearing tumor with ICAM-1 knockdown had a prolongation of overall survival compared to control tumor bearing mice. These data indicate that targeting ICAM-1 would be a potentially powerful approach in controlling glioma progression in patients with recurrent glioblastoma refractory to antiangiogenic therapy. Such an approach is supported by previous studies which have shown that vaccination with ICAM-1–transfected tumor cells markedly inhibited the growth of subcutaneously inoculated glioma but not glioma located in the brain [7]. The ICAM-1 monoclonal antibody UV3, an anti–human CD54 antibody, has also been found to inhibit the growth of multiple myeloma, lymphoma, uveal melanoma, breast cancer, prostate cancer, non–small cell lung cancer, and pancreatic cancer in severe combined immunodeficiency mice [7]. One recent study also demonstrated that the BI-505 antibody, an anti–human ICAM-1 antibody, could specifically target B-cell tumors and had broad anti-myeloma activity *in vivo* by inducing programmed cell death [10]. These findings suggest that ICAM-1 is potentially an important target for immunotherapy.

The present study’s findings also provide new insights into ICAM-1 expression, specifically as it occurs under hypoxic conditions. Previous studies have shown that ICAM-1 is highly expressed in glioblastoma, only weakly expressed or absent in low-grade glioma, and undetectable in normal and fetal brain [11]. ICAM-1 expression is regulated by cytokines or growth factors, including tumor necrosis factor-α, interferon-γ, interleukin-1β, and transforming growth factor-β [11]. Our immunohistochemical analysis revealed that ICAM-1 is expressed only in the hypoxic areas of GSC11 xenografts from mice treated with antiangiogenic therapy. Accumulated data have shown that chronic antiangiogenic therapy leads to excessive pruning of tumor vessels, promoting hypoxia. Both hypoxia and antiangiogenic therapy are known to promote epithelial-to-mesenchymal transition in several epithelial tumors [8]. In most human cancers, including malignant glioma, HIF-1α expression promotes tumor growth, angiogenesis, and disease progression [12]. Hypoxia and HIF-1α have been associated with radiotherapy and chemotherapy resistance. We found that ICAM-1 expression was up-regulated by p-STAT3 under hypoxic conditions *in vivo* and *in vitro*. In addition to HIF-1α, many other nuclear transcription factors have also been shown to regulate ICAM-1 gene expression, including activator protein-1, nuclear factor-κB, CCAAT/enhancer binding protein, E26 transformation-specific transcription factor, STAT, and specificity protein-1 [13]. The ICAM-1 interferon-γ response element is a palindromic STAT binding site and is homologous to interferon-γ–activating sequences [14]. A more recent study showed that ICAM-1 expression in glioma was upregulated by the activation and interaction of nuclear factor-κB and STAT3 and mediated tumor cell invasion and migration in response to radiotherapy [15]. Carro et al. implanted SNB75 glioma cells into nude mice, extracted RNA from the tumor site, and subjected it to a chromatin immunoprecipitation assay and found that STAT3 binds to ICAM-1 [16]. In the present study, we found that inhibiting STAT3 blocked ICAM-1 expression under hypoxic conditions *in vitro*.

The association of ICAM-1 with invasion and metastases has been demonstrated in several types of cancer. Tumors’ resistance to antiangiogenic therapy is facilitated by cell invasion and metastasis, which provides tumor cells with access to normal vasculature [17, 18]. Notably, ICAM-1 overexpression has been associated with more aggressive tumor phenotypes and poor prognosis in different types of malignancies, including multiple myeloma [19], breast cancer [20], gastric cancer [21], liver cancer [22], and pancreatic cancer [23]. Rosette et al. reported that downregulation of ICAM-1 at the mRNA and protein levels strongly suppressed the invasion of human breast cancer cells *in vitro* [24]. Previously, we found that antiangiogenic therapy might promote glioma cell invasion by promoting the mesenchymal transition and invasive capacity of glioma cells [9]. In the present study, we found that inhibiting ICAM-1 expression reduced glioma invasion *in vivo* and *in vitro*, suggesting a potentially important role for ICAM-1 in glioma invasion.

In the present study, we found that ICAM-1 knockdown reduced macrophage infiltration into tumor *in vivo* and that an anti–ICAM-1 antibody inhibited tumor cells from migrating to macrophages *in vitro*. We also found that ICAM-1 was related to the glioma-associated macrophages involved in the glioma cells’ resistance to antiangiogenic therapy. These findings are in line with those of other studies, which have shown that myeloid-derived cells (the precursors of monocytes, macrophages, and granulocytes) mediate resistance to antiangiogenic therapy [25]. Shojaei et al. found that therapy resistance was associated with a significant increase in tumor-infiltrating myeloid cells, which were capable of promoting angiogenesis independent of VEGF [25]. Tumors can direct the bone marrow to increase myeloid cell production and enhance the recruitment of these cells to the tumor [26]. In this process, ICAM-1 may play an important role in regulating myeloid cell migration by binding with LFA-1 and Mac-1, both of which are expressed in leukocytes and promote the adhesion and transendothelial migration of leukocytes. The adhesion of monocytes to the vessel wall is mediated by adhesion molecules such as integrins and ICAM-1 [27]. Interestingly, Zheng et al. found that ICAM-1/CD18 played an important role in myeloma cells’ macrophage-mediated resistance to chemotherapy [19].

In conclusion, we demonstrated in this study that ICAM-1, which is induced through pSTAT3 activation under hypoxic conditions, promotes glioma cell invasion and tumor progression; knocking down ICAM-1 expression inhibited tumor growth in and prolonged the overall survival of glioma-bearing mice; and knocking down ICAM-1 resulted in less macrophage infiltration and a reduction in tumor invasion. Together, these results suggest that targeting ICAM-1 could be a useful approach in overcoming glioblastoma’s resistance to antiangiogenic therapy, thereby improving the prognosis of patients with this disease.

## MATERIALS AND METHODS

### Cell lines and reagents

The glioma stem cell lines GSC6-27, GSC7-2, GSC11, GSC17, GSC231, GSC20, GSC272, GSC28, GSC8-11, GSC23, and GSC280 were obtained from Drs. Howard Colman, Erik Sulman, and Frederick Lang (The University of Texas MD Anderson Cancer Center, Houston, TX) [28]. Glioma stem cell development is funded by the MD Anderson Brain Cancer SPORE supported by P50CA127001.GSC lines were isolated from fresh surgical specimens of human glioblastoma and cultured as glioblastoma neurospheres in Dulbecco’s Modified Eagle Medium (DMEM/F12) containing EGF (20 mg/ml), basic fibroblast growth factor (bFGF) (20 mg/ml), and B27 (2%) at 37°C in 5% CO_2_ atmosphere. Acquisition of these cell lines was approved by the Institutional Review Board of MD Anderson Cancer Center and were obtained from 2005 to 2012. The macrophage (American Type Culture Collection) were maintained in RPMI 1640 medium supplemented with 10% fetal bovine serum and penicillin/streptomycin.

LY294002, SU6656 and U01260 were purchased from Selleckchem. AZD1480 was provided by AstraZeneca.

### Transfection

Cells were plated at a density of 3 × 10^5^ per six-well plate. Transfection was carried out using HyFect reagents (Deveville Scientific) according to the vendor’s instructions. Transfected cells were selected with puromycin (5 mg/ml) for 10–14 days. At that time, antibiotic-resistant colonies were picked, pooled, and expanded for further analysis. The PGIPZ control was generated with control oligonucleotide GFTTCTAACACCGGAGGTCTT. PGIPZ ICAM1#2 shRNA was generated with 5′ - GCATTAAAGCAGCGTATC - 3′ and PGIPZ ICAM1#4 shRNA was generated with 5′ - GCATTAAAGCAGCGTATC - 3′.

### Immunoblotting analysis

Cells were lysed in an ice-cold lysis buffer containing 50 mM Tris-Cl, pH 7.5, 100 mM NaCl, 1 mM EDTA, 1% TritonX-100, 1 mM PMSF, 1 μg/ml leupeptin, and 1 μg/ml pepstain A. The protein concentration in the supernatant was determined using a BCA protein assay (Pierce, Rockford, IL, USA). Samples were subjected to 8–12% SDS-polyacrylamide gel electrophoresis, and the separated proteins were electrophoretically transferred to nitrocellulose membranes. Blots were incubated with the primary antibody against ICAM-1 (1:1000; Cell signaling Technology, Danvers, MA), GFP (1:1000, CST), p-STAT3 (1:1000, Cell signaling), Tubulin (1:3000; Sigma), p-AKT (1: 1000, Cell signaling Technology, Danvers, MA), AKT (1: 1000, Cell signaling). The membranes were then incubated with horseradish peroxidase-linked secondary anti-rabbit or anti-mouse antibodies (Bio-Rad).

### Animal xenografts and treatment

For the *in vivo* experiments, we used 4 - to 6-week –old female nude mice strictly inbred at the University of Texas MD Anderson Cancer Center (MD Anderson) and maintained in the MD Anderson Isolation Facility in accordance with Laboratory Animal Resources Commission standards and treated according to an approved protocol. GSC11 and shICAM-1 #4 cells (3 × 10^5^) were implanted intracranially into nude mice. Four days after implantation, bevacizumab (10 mg/kg) or vehicle was administered by intraperitoneal injection twice a week. When the mice developed signs and symptoms of advanced tumor growth, they were compassionately euthanized with CO_2_ in accordance with animal welfare guidelines. The mice’s brains were then removed and fixed in 4% formaldehyde, and embedded in paraffin. Tumor formation and the phenotype were determined by histologic analysis of H&E-stained sections. In order to determine tumor volume by external caliper, the greatest longitudinal diameter (length) and greatest transverse diameter (width) were determined. Tumor volumes were based on computer caliper measurements of digitized whole mount coronal CNS sections at the tumor injection site and were calculated by the modified ellipsoidal formula: Tumor volume = 12 (length × width2) [29].

### Immunohistochemistry and immunofluorescence

Brain tissues were fixed in 4% paraformaldehyde for 24 hours, embedded in paraffin, serially cut into 4-µm sections, and stained with H&E (Sigma-Aldrich, St.Louis, MO, USA). For immunohistochemical stains, the tumor sections were deparaffinized and subjected to graded rehydration. After blocking the sections in 5% serum and performing antigen retrieval (citrate buffer, pH 6.0), we incubated the sections with the primary antibodies overnight at 4°C. Immunohistochemistry and immunofluorescence staining was performed using the primary antibody against ICAM-1 (1:100; Cell signaling Technology, Danvers, MA), Nestin (1:500; Millipore, Billerica, MA), F4/80 (1:50; Biolegend, San Diego, CA) and HIF-1 alpha (1:100; Novus Biological, Littleton,CO).We then incubated the sections with Texas red fluorescein isothiocyanate–conjugated secondary antibodies and green fluorescein isothiocyanate–conjugated secondary antibodies (Invitrogen, Grand Island, NY) for 1 hour at room temperature.

### Invasion assay

Matrigel basement membrane matrix (BD labware, New Jersey, USA) was used to conduct the *in vitro* cell invasion assays. Transwell inserts with 8-µm pores were coated with 100 µl of Matrigel in cold serum-free medium at a final concentration of 0.35 mg/ml. The GSC11, shICAM-1 #2 and shICAM-1 #4 cells suspension (2 × 10^5^ cells in 100 µl of medium) were added to the transwell inserts in triplicate. Cells were allowed to invade for 24 hours at 37°C. The filters were then fixed and stained with 0.1% crystal violet in 20% methanol. The invasive cells were visualized using bright-field microscopy. Transwell membranes were incubated with 4% deoxycholic acid for 20 minutes, and the absorbance was read at 595 nm.

### Real-time PCR

Total RNA was extracted from tumor-bearing mouse brain using the RNeasy Mini Kit (Qiagen, Valencia, CA, USA ) coupled with DNase treatment and then reverse-transcribed with the High Capacity cDNA Reverse Transcription Kit (Applied Biosystems, Grand Island, NY,USA) according to the manufacturers’ instructions Each cDNA was analyzed in triplicate using real-time TaqMan probes (Applied Biosystems, Grand Island, NY,USA). Quantitative PCR analysis was conducted using the Chromo4 sequence-detection system (Bio-Rad, Hercules, CA, USA). Relative quantification of mRNA levels was conducted using the comparative Ct method with GADPH as the reference gene and the formula 2−ΔΔCt.

### Cell binding assay

A cell binding assay was performed as described previously [30]. Macrophage 2 × 10^5^ were seeded on glass coverslips. After 24 hours, the GSC11 cells were seed on same well treated with or without with ICAM-1 antibody. After incubation for 2 hour 37°C, the wells were gently washed twice with medium to remove non-adherent cells. The Macrophage were stained with F4/80 (1:50; Biolegend, San Diego, CA), and GSC11 cells were stained with stem cell marker Nestin (1:300; Abcam, Cambridge, MA). The ratio of adherent macrophage cells to GSC11 cells was calculated as the ratio of F4/80 positive cells to Nestin positive cells.

### Statistical analysis

All statistical analyses were conducted with the GraphPad (InStat) software 6 for Windows 7. Survival analysis was conducted using the Kaplan–Meier method, and differences between cohorts were assessed using the log-rank test. All other data were compared using an unpaired two-tailed Student *t*-test. Summary statistics for continuous data are expressed as the mean ± standard error of the mean. *P* values less than 0.05 were considered statistically significant. The Nestin and ICAM-1 staining was calculated by using the Image-Pro Plus system version 7.0 (Media Cybernetics) in × 10 fields of at least three tumor samples per group and three to four different section per tumor sample.

## SUPPLEMENTARY MATERIALS FIGURES


