# Supplementary material for: Targeting intercellular adhesion molecule-1 prolongs survival in mice bearing bevacizumab-resistant glioblastoma
Source: Oncotarget. 2017 Jun 29;8(57):96970–83. doi: 10.18632/oncotarget.18859 (PMC5722538; doi:10.18632/oncotarget.18859)
Supplement: Supplementary file 1 [file oncotarget-08-96970-s001.pdf]

Targeting intercellular adhesion molecule-1 prolongs survival in mice bearing bevacizumab-resistant glioblastoma

Supplementary Materials

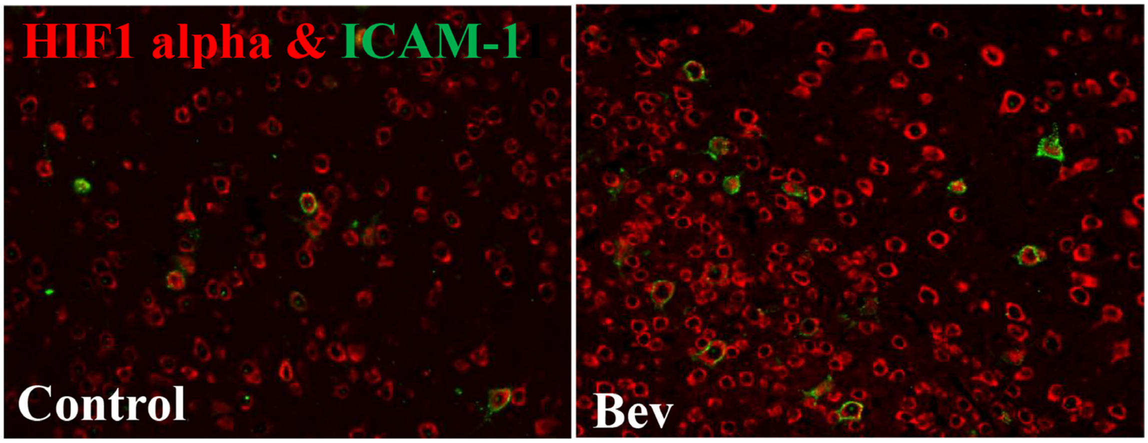

Supplementary Figure 1: Immunofluorescence staining with HIF-1 (red) and ICAM (green) was performed. Data revealed that ICAM1 and HIF-1 were overexpressed in bevacizumab resistant tumor.

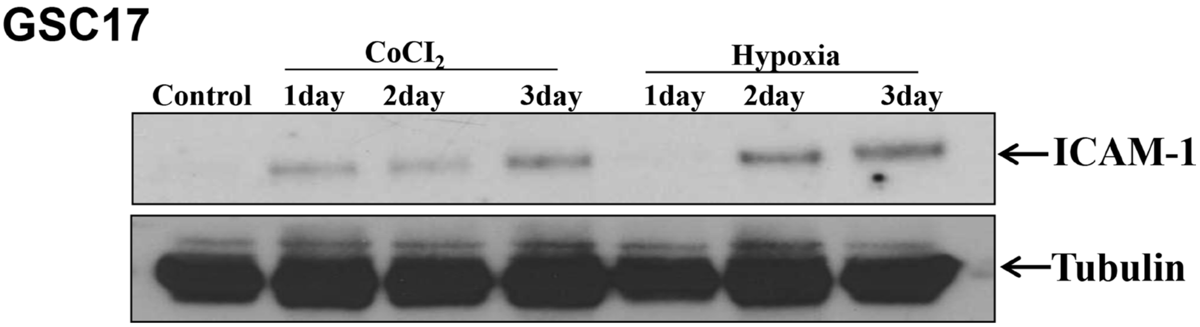

Supplementary Figure 2: Western blotting revealed that the ICAM-1 expression level in GSC17 cells was increased in a time-dependent manner under hypoxic conditions.

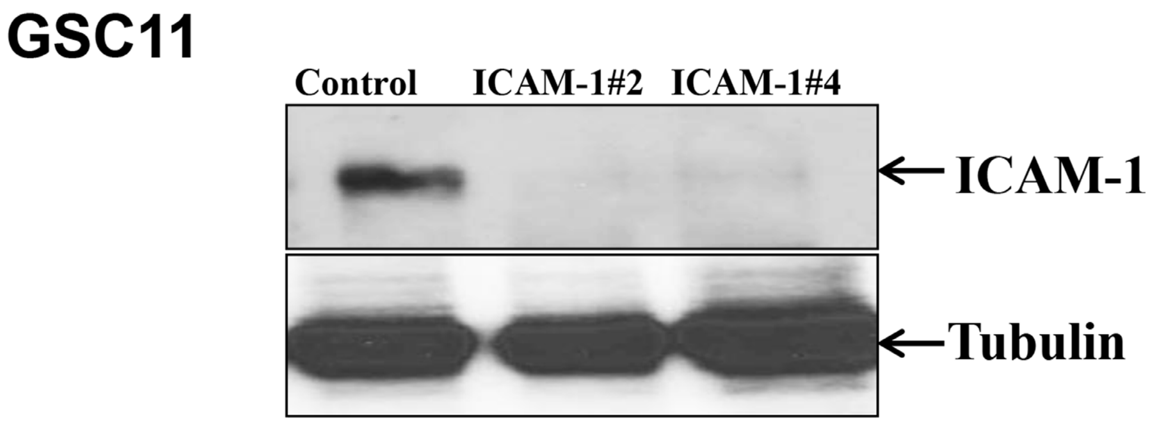

Supplementary Figure 3: Western blotting revealed that the ICAM-1 expression level in GSC11 was lower in the cells which were transfected with shRNA ICAM-1 #2 and #4 constructions.
